# Association between Green Tea Consumption and Abdominal Obesity Risk in Middle-Aged Korean Population: Findings from the Korean Genome and Epidemiology Study

**DOI:** 10.3390/ijerph19052735

**Published:** 2022-02-26

**Authors:** Junkyung Kwak, Dayeon Shin

**Affiliations:** Department of Food and Nutrition, Inha University, Incheon 22212, Korea; oppo9012@naver.com

**Keywords:** metabolic syndrome, abdominal obesity, green tea consumption, Korean Genome and Epidemiology Study (KoGES)

## Abstract

The prevalence of general and abdominal obesity is increasing with rapid economic growth and the westernization of dietary habits in Korea, especially in the middle-aged population. Data were obtained from the Korean Genome and Epidemiology Study (KoGES), which recruited 10,030 participants between the ages of 40 and 69 years. Information on green tea consumption was obtained from the food frequency questionnaire and categorized as none, <1 cup, between 1 and <4 cups, and ≥4 cups. Multivariable logistic regression models were used to estimate the ORs and 95% CIs to examine any possible associations between green tea consumption and the risk of abdominal obesity after controlling for potential confounders. High consumption of green tea was associated with a 44% lower odds ratio for abdominal obesity (none vs. ≥4 cups/week: OR, 0.56; 95% CI 0.41-0.78; *p* for trend = 0.001). When stratified by sex, an inverse association between green tea consumption and abdominal obesity was observed only in women (none vs. ≥4 cups/week: OR, 0.71; 95% CI 0.57–0.88; *p* for trend = 0.004). No significant association was found among men. Our findings indicate that green tea consumption has beneficial effects in the prevention of abdominal obesity in middle-aged Korean women.

## 1. Introduction

Abdominal obesity is a metabolic syndrome that is highly associated with factors that significantly increase the risk of cardiovascular disease (CVD) [1]. According to the Statistical Yearbook of Health Examinations published in 2019 by the National Health Insurance Corporation, the overall prevalence of abdominal obesity in Koreans is 24.8% (29.5% in men and 19.8% in women). The overall prevalence of abdominal obesity of people under the age of 20 was 15.3% (22.0% in men and 8.4% in women), whereas the overall prevalence among people in their 50s was 23.4% (28.2% in men and 18.6% in women) [2]. According to a survey, the prevalence of abdominal obesity was approximately 10% higher in those aged 40–69 years. Abdominal obesity was independently associated with CVD, myocardial infarction, and total mortality [3,4,5]. An effective reduction in waist circumference results from reduced abdominal fat and body fat-related risk rates, such as serum lipids [1,6]. Abdominal fat is influenced by environmental factors such as physical activity, genetic factors, sex, hormones, stress, and dietary quality, which are risk factors for abdominal obesity [7,8]. Many studies have shown that the dietary factors from among these various risk factors are particularly significant and directly contribute to the abdominal obesity rates [9,10,11].

Green tea is mainly consumed in Asia and has various pharmacological effects, including the treatment and prevention of obesity, particularly because it is associated with increased fatty acid oxidation and reduces body fat and abdominal fat [6,12,13,14,15]. An experimental epidemiological study conducted in Japan reported that weight gain and fat tissue in the abdominal cavity were significantly suppressed in the diet groups containing 2% and 4% powdered green tea as a result of supplying a diet containing 1%, 2%, and 4% powdered green tea [16]. Furthermore, an analysis of total cholesterol in the liver, neutrophils in serum and liver, free fatty acid (FFA) in serum, and leptin levels showed that the powdered green tea group had lower concentrations than the control group in 4-week-old ICR female mice (18 g) [16]. Another experimental study conducted in Japan reported that consumption of green tea catechins (625 mg/day) enhanced exercise-induced loss of abdominal fat and was associated with decreased triglyceride levels and the circulating FFA in overweight and obese adults [17]. An experimental epidemiological study conducted in China reported that incubation with green tea polyphenols (GTPs) promoted catabolism of abdominal adipose tissue and was associated with increased lipid metabolism in the skeletal muscle of male chickens (35 days old) [18]. A randomized, placebo-controlled study conducted in Shanghai reported that daily consumption of 500–900 mg of green tea catechin with less than 200 mg of caffeine can have a positive effect on reducing abdominal fat, particularly in Asian and overweight subjects [19]. Several animal and human studies reported that the groups that consumed green tea were significantly associated with decreased blood glucose, total cholesterol, LDL cholesterol levels, and triglycerides while they were associated with increased HDL cholesterol levels compared to the control group [14,15,20,21]. 

In Korea, with rapid economic growth and westernization of dietary habits, the prevalence of abdominal and general obesity is increasing, especially in the middle-aged population. However, most studies have been conducted on the role of green tea consumption in general obesity, and few studies have focused on the prevalence of abdominal obesity in Korean adults. In this study, the association between green tea consumption and abdominal obesity was examined using data derived from the Korean Genome and Epidemiology Study (KoGES) cohort of middle-aged Korean adults. Furthermore, the characteristics of the population, based on abdominal obesity and green tea consumption, were investigated. Abdominal obesity is a component of metabolic syndrome and is known to be related to other biomarkers such as blood pressure, fasting blood glucose, and blood lipid levels [1]; therefore, this study analyzed it with blood indicators related to abdominal obesity. 

## 2. Materials and Methods

### 2.1. Study Participants

The KoGES, a large, population-based prospective cohort study, targeted Koreans with chronic diseases such as hypertension, obesity, cardiovascular disease, metabolic syndrome and type 2 diabetes [22]. It was designed to identify genetic and environmental factors and their interrelationships [22]. Since 2001, studies have been conducted in the Ansan and Ansung regions of South Korea on the KoGES. The Ansung cohort consisted of a rural population, while the Ansan cohort was a medium-sized city population.

**Figure 1:**
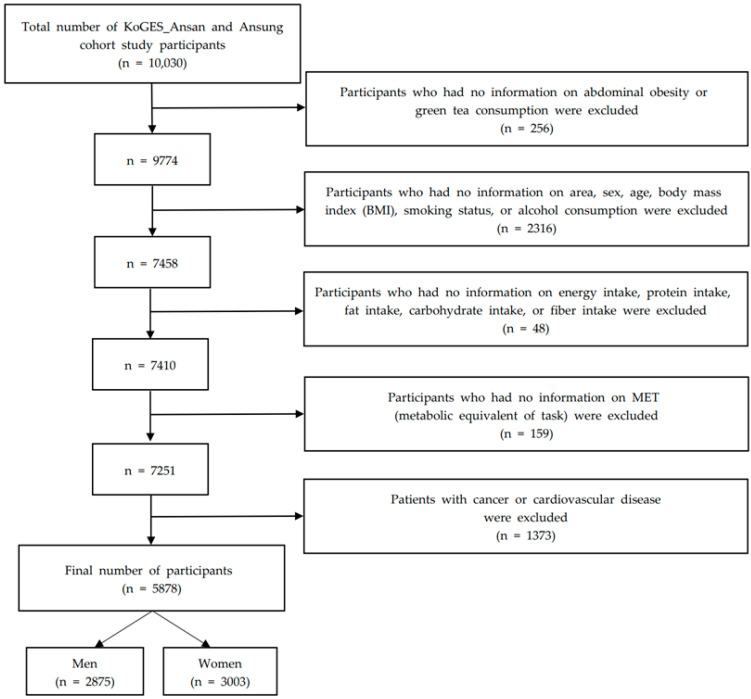
Process flow chart outlining the relevant steps for this analysis.

In brief, 10,030 participants were enrolled between the ages of 40 and 69 (5012 from a farming community in Ansung and 5018 from an urban community in Ansan). Of these 10,030 participants, those who did not provide information on waist circumference measurements and green tea consumption were excluded (n = 256, 2.6%). Among the remaining 9774 participants, those with missing information on area, sex, age, BMI, smoking status, and alcohol consumption were excluded (n = 2316). Among the remaining 7458 subjects, participants with missing information on fiber intake, carbohydrate intake, fat intake, protein intake, and energy intake were excluded (n = 48). Among the remaining 7410 subjects, participants who had missing information were excluded (n = 159). Among the remaining 7251 subjects, those with a history of cancer and CVD (treatment history, drug history, and medical history of hypertension, coronary artery disease, congestive heart failure, myocardial infarction and hyperlipidemia) were excluded (n = 1373) [23]. The final analytic sample size consisted of 5878 subjects (2875 men and 3003 women) according to the exclusion criteria of study participants (Figure 1) [24].

### 2.2. Assessment of Abdominal Obesity

Waist circumference was measured using standard methods with light clothing. It was calculated as the average of measurements over three times with the perimeter horizontal, the intermediate point between the rib (rib bone) and the iliac ridge. The average of measurements was read up to one decimal point in centimeters (cm). Abdominal obesity is defined as at least 90 cm around the waist of a man and, 80 cm around the waist of a woman, as defined by the amended National Cholesterol Educational Program Adult Treatment Panel III (NCEP-ATP III) [1]. 

### 2.3. Assessment of Green Tea Consumption

The participants’ green tea intake was estimated based on a semi-quantitative food frequency questionnaire (FFQ) comprising 106 food items. This questionnaire was developed to determine the relationship between dietary intake and disease among Koreans [25]. Using a questionnaire based on foods frequently consumed by Koreans aged 40–69 years, a food list and reference amount were presented to investigate the frequency and amount of intake of each food, and various auxiliary tools were used so that the subjects could accurately respond to the amount of food consumed. The participants’ usual green tea intake was investigated using a semi-quantitative FFQ during the past year. Follow-up was conducted to complete the FFQ survey periodically by well-trained interviewers. Green tea consumption was surveyed based on a green tea bag sold in the market. The amount of green tea served (120 mL) was set based on the reference amount suggested by the KoGES. Green tea intake in the questionnaire was classified according to the frequency of intake (1–2 times a day, 3–4 times a day, and 5 times a day) and average intake per serving (1/2 cup, 1 cup, and 2 cups). In this study, green tea intake was classified according to frequency (none, <1 cup, between 1 and <4 cups, and ≥4 cups) on a weekly basis, and the portion size was set at 120 mL per cup based on the KoGES FFQ guideline [26]. 

### 2.4. Assessment of Other Variables

KoGES research data collection was conducted only for participants who showed a willingness to participate in the study. To determine the demographic information, lifestyle, medical history, and health conditions of the study participants, a survey and examination were conducted for 2–3 h at the site. Blood and urine were used for biochemical tests to diagnose health conditions, and the rest were separated according to their components (serum, plasma, DNA, etc.) and stored at the Korea Centers for Disease Control and Prevention. 

Education level was classified as elementary school or lower, middle/high school, and college or higher. Household income was categorized as <100, 100–200, 200–300, and >300 (10,000 won/month). The MET, which is based on physical activity during the day, was classified as follows: sedentary lifestyle, low-intensity activity, moderate-intensity activity, and high-intensity activity. Specifically, sedentary life included typing, driving, office work, playing, sewing class, ironing, writing, and cooking; low-intensity activities included walking, cleaning, laundry, babysitting, bathing, and exercise as entertainment (bicycle, table tennis); medium-intensity activities included fast walking, woodwork, lawn mowing, snow removal, and regular exercise (badminton, swimming, tennis); and high-intensity activities included sports, mountaineering, running, tree cutting, agriculture, forestry, and mining. After entering different values according to exercise intensity, the MET was calculated by changing the existing units of minutes per day to hours per day. 

The follow-up participants regularly attended community clinics for anthropometric measurements and biochemical tests (including fasting blood glucose, fasting insulin, C-reactive protein (CRP), blood pressure, blood lipid levels, waist circumference, weight, and height) performed by trained healthcare providers. BMI was calculated as the weight divided by height squared (kg/m^2^) and was measured using tetrapolar bioelectrical impedance analysis (InBody version 3.0; InBody, Seoul, Korea). After overnight fasting for 12 h, the plasma concentrations of total cholesterol, HDL cholesterol, glucose and triglycerides were assessed using an immunoradiometric assay (ADVIA 1650; Siemens, Tarrytown, NY, USA). Systolic and diastolic blood pressures were measured on the left and right arms with the subjects in a sitting position in a stable state, and their average values were calculated. When measuring blood pressure, the arm and back were supported, and blood pressure was assessed in a sitting posture with the arm held at the heart level. 

In the FFQ, the intake of nutrients of each food item was calculated, taking into account the weight caused by the portion size of each food item and the frequency of intake. The sum of the nutrients consumed by each food item determines the daily nutrient intake of the individual. Total energy intake was calculated in kcal per day (kcal/day). Carbohydrate intake, protein intake, fat intake, and fiber intake were calculated in grams per day (g/day).

### 2.5. Statistical Analyses

All data are expressed as mean and standard deviation or as numbers and percentages for descriptive statistics. The general characteristics of the participants according to abdominal obesity were compared using the chi-squared test for categorical variables and *t*-test for continuous variables. The general characteristics of the participants according to weekly green tea consumption were compared using chi-squared tests for categorical variables and ANOVA for continuous variables. 

The relationship between green tea consumption and abdominal obesity was examined using multiple logistic regression models. Unadjusted and adjusted odds ratios (ORs) and 95% confidence intervals [27] were calculated to assess the association between green tea consumption and abdominal obesity. The *p*-value for trend was calculated using the median for each category of weekly green tea consumption as a continuous variable. Adjustments were made for age, sex, area, alcohol consumption, smoking, BMI, education level, household income, MET, energy intake, protein intake, fat intake, carbohydrate intake, and fiber intake. We further analyzed the effects of green tea consumption by sex because the prevalence of abdominal obesity and reported risk factors differ by sex. All statistical analyses were performed using IBM SPSS (Statistical Package for Social Science) Statistics 26 (IBM, Armonk, NY, USA). Statistical significance was set at *p* < 0.05.

## 3. Results

### 3.1. Associations of Abdominal Obesity with Other Risk Factors

Table 1 describes the general characteristics of the participants according to sex and abdominal obesity (women with abdominal obesity, n = 1367; women without abdominal obesity, n = 1636; men with abdominal obesity, n = 520; men without abdominal obesity, n = 2355). 

Regardless of sex, the abdominal obesity group had a higher proportion of residents in Ansung, BMI (kg/m^2^), fasting blood glucose (mg/dL), total cholesterol (mg/dL), triglyceride levels (mg/dL), CRP (mg/dL), fasting insulin (uIU/mL), average systolic blood pressure (mmHg), average diastolic blood pressure (mmHg), total energy intake (kcal/day), carbohydrate intake (g/day), and fiber intake (g/day) and lower HDL cholesterol (mg/dL) compared to the non-abdominal obesity group (all *p* values < 0.05). In women, the abdominal obesity group had a higher mean age, MET (hours/day), the proportion of education level of elementary school or lower, income level less than 100 (10,000 won/month), and non-drinkers and lower fat intake (g/day) compared to the non-abdominal obesity group (all *p* values < 0.05). Among men, the abdominal obesity group had higher protein intake (g/day) and fat intake (g/day) and lower MET (hours/day) than the non-abdominal obesity group (all *p* values < 0.05). Regarding the relationship between abdominal obesity and weekly green tea consumption (in cups), weekly green tea consumption was higher in the non-abdominal obesity group than in the abdominal obesity group only in women (*p* < 0.0001; 1.8 ± 4.3 vs. 2.6 ± 4.9).

### 3.2. Associations of Green Tea Consumption per Week with Other Risk Factors

Table 2 describes the participants’ general characteristics according to sex and weekly green tea consumption (none, n = 2236; <1 cup/week, n = 1058; 1 to <4 cups/week, n = 1501; ≥4 cups/week, n = 1083). 

Regardless of sex, frequent green tea drinkers (≥4 cups/week) had lower mean age, MET, proportion of residents in Ansung, education level of elementary school or lower, income level less than 100 (10,000 won/month), average systolic blood pressure (mmHg), total energy intake (kcal/day), protein intake, fat intake (g/day), carbohydrate intake (g/day), and fiber intake (g/day) compared with non-drinkers (all *p* values < 0.05). In women, frequent green tea drinkers (≥4 cups/week) had a lower proportion of non-drinkers, total cholesterol (mg/dL), HDL cholesterol (mg/dL), and average diastolic blood pressure (mmHg) than non-drinkers (all *p* values < 0.05). In men, frequent green tea drinkers (≥4 cups/week) had higher BMI, proportion of non-smokers, fasting blood glucose (mg/dL), and total cholesterol (mg/dL) than non-drinkers (all *p* values < 0.0001).

### 3.3. Risk of Abdominal Obesity Due to Green Tea Consumption

To assess the prevalence of abdominal obesity in relation to green tea consumption, multivariable logistic regression analysis was performed according to the level of green tea consumption per week to compute the adjusted odds ratio and 95% CI, as shown in Table 3. We adjusted for age, sex, area, alcohol consumption, smoking, BMI, education level, household income, MET, energy intake, protein intake, fat intake, carbohydrate intake, and fiber intake. Compared with participants who did not drink green tea regardless of sex, crude ORs for the prevalence of abdominal obesity were 0.73 (95% CI: 0.60–0.90), 0.52 (95% CI: 0.43–0.63), and 0.45 (95% CI: 0.36–0.56) for those drinking <1 cup/week, 1 to <4 cups/week, and ≥4 cups/week, respectively. 

After adjusting for confounding variables, multivariable ORs were significantly associated with a decrease for those drinking <1 cup/week (OR: 0.89; 95% CI: 0.67−1.18), 1 to <4 cups/week (OR: 0.74; 95% CI: 0.56–0.98), and ≥4 cups/week (OR: 0.56; 95% CI: 0.41−0.78) compared to those who did not drink green tea, regardless of sex. Based on the participants who did not consume green tea among women, participants that consumed 1 to <4 cups/week or ≥4 cups/week of green tea had crude ORs (95% CI) for abdominal obesity of 0.72 (0.64−0.81) and 0.65 (0.56−0.74), respectively. Furthermore, women who consumed 1 to <4 cups/week or ≥4 cups/week of green tea had multivariable-adjusted OR (95% CI) for abdominal obesity of 0.79 (0.65−0.96) and 0.71 (0.57−0.88), respectively, after adjusting for age, area, alcohol consumption, smoking, BMI, education level, household income, MET, energy intake, protein intake, fat intake, carbohydrate intake, and fiber intake. When stratified by sex, the degree of the association between green tea consumption and the prevalence of abdominal obesity in women tended to be significantly lower. 

## 4. Discussion

In this study, we investigated the beneficial effects of green tea consumption on abdominal obesity according to sex, using community-based cohort data of middle-aged Korean adults. We found a significant negative correlation between the total population and women, but not for men as a result of studying the associations between abdominal obesity and green tea consumption. 

In Ansung, a rural area, the proportion of participants who did not consume green tea was the highest, and in Ansan, an urban area, the proportion of participants who consumed more than four cups of green tea per week was the highest. In a survey of green tea consumers in Korea, China, and Japan, 216 Koreans answered that health was their motivation for purchasing green tea (n = 80, 37.0%), with the highest proportion of participants consuming it at home (n = 99, 45.8%) or at an office/school (n = 81, 37.5%) [28]. In a result of a survey of consumers’ perceptions of green tea in Daegu, Korea, 287 out of 296 participants answered that they drink it because it is good for health [29]. According to the 2008–2019 Community Health Survey conducted by the Korean Statistical Information Service (KOSIS), the health behavior practice rate in Ansan, a large city, was 33.2% (n = 1822), while that in Ansung, a rural area, was 23% (n = 908) [30]. There was a difference of approximately 10%, depending on the scale of the residential area. This disparity according to a residential area may be due to a difference in the perception of health depending on whether the residential area is urban or rural [31,32]. Additionally, health inequalities depending on the area are one of the socioeconomic factors contributing to the difference in healthy behavior practice rates [31,33]. Differences in health perceptions may have affected the consumption of green tea. The results of this study also showed health inequality in terms of education and income levels, lower consumption of green tea per week, and lower education and income levels. Area, education level, and income level are all socioeconomic factors, indicating that one group has a lower average health level than others [34]. As the standard of living in the 21st century has enhanced overall, the health gap is gradually narrowing, but since socioeconomic differences in Korea have an impact on the risk of abdominal obesity, they are worth paying attention to [34,35,36]. 

Drinking and smoking based on the amount of weekly green tea consumption, tended to be inversely associated, depending on sex. The proportion of current drinkers was higher in the group that consumed ≥4 cups/week of green tea than in green tea non-drinkers in men and women. In contrast, the proportion of current drinkers was higher in green tea non-drinkers than in men who consumed ≥4 cups/week of green tea. Alcoholic ethanol oxidation is accompanied by free radical production, and green tea acts as an antioxidant in the liver and serum by preventing ethanol-induced changes [37,38]. This antioxidant effect of green tea is thought to suppress the risk of abdominal obesity in women who consume alcohol. Green tea extracts reduce the level of oxidative stress in smokers [39]. According to cell culture and animal model studies, EGCG, a major polyphenol in green tea, has strong anti-inflammatory and anti-proliferative activities that can selectively inhibit cell growth and induce apoptosis in cancer cells, thus interfering with the adverse effects of tobacco smoke on various organs (bronchial epithelial cells, lungs, and liver) [40,41,42]. Furthermore, green tea consumption may have a positive effect on the risk of abdominal obesity in both smokers and non-smokers who may be passively exposed to smoke by reducing the level of oxidative stress. In summary, green tea acts as an antioxidant by lowering the oxidative stress caused by smoking and drinking, which reduces their harmful effects and further reduces the risk of abdominal obesity [43]. 

In this study, higher consumption of green tea was significantly associated with decreased total cholesterol and increased HDL cholesterol levels in women. In men, higher consumption of green tea was associated with significantly increased fasting blood glucose and total cholesterol levels. Many studies have demonstrated that green tea consumption is significantly associated with blood indicators [15,20,44,45,46]. In a case-control study of Sprague–Dawley male rats (180–200 g) in Korea, a combination of GTPs and vitamin C was more effective in reducing blood glucose, insulin resistance, and serum triglycerides than either substance administered alone [47]. In another experimental epidemiological study of male mice aged 4 weeks in Korea, EQS mixed with green tea extract, dietary fiber (polydextrose), and vitamin C was demonstrated to be highly effective against oxidative stress in obesity [48]. Green tea consumption is related to blood indicators; therefore, it is helpful not only in abdominal obesity but also in cardiovascular diseases such as dyslipidemia, arteriosclerosis, diabetes, and general obesity [15,49,50,51]. The relationship between total cholesterol and HDL cholesterol levels and green tea intake in women was consistent with previous studies, but the relationship between fasting blood glucose and total cholesterol levels and green tea intake in men was not explained by the results of previous studies [52,53].

In women, the higher the amount of green tea consumption, the more significantly lower the systolic and diastolic blood pressures, and in men, the higher the amount of green tea consumption, the more significantly lower the systolic blood pressure. The antioxidant properties of GTPs help to inhibit higher blood pressure and have a positive effect on the treatment of hypertensive patients by lowering blood pressure indicators [54,55]. High blood pressure is strongly associated with abdominal obesity, which is a predictor of future hypertension [56]. Both women and men tended to have significantly higher total energy, carbohydrate, and fat intakes and higher green tea consumption. High-carbohydrate and high-fat diets are associated with the risk of abdominal obesity [57,58]. In many experimental epidemiological studies that demonstrated the efficacy of green tea, most mice and rats were fed a high-fat diet to induce obesity and a positive effect was observed on blood indicators and lipid metabolism with regard to insulin resistance [45,49,59,60]. It has been determined that a high-fat diet intake causes abdominal obesity, but not all dietary fat accumulates as abdominal fat [61,62]. It is also necessary to study the association between the type of dietary fat and green tea consumption through experimental studies on human subjects.

Both women and men tended to have significantly higher dietary fiber and protein intakes with higher green tea consumption. In contrast to total energy, carbohydrate and fat intake, fiber and protein intake were linked to a reduced risk of abdominal obesity [63,64,65,66]. In Korea, various experimental epidemiological studies on high-fat diet-induced Sprague–Dawley rat obesity have confirmed the antioxidant and anti-inflammatory effects of dietary fiber added to green tea, which have a positive effect on abdominal obesity through the reduction of abdominal fat [21,48]. 

Among the ingredients of green tea, epigallocatechin-3-gallate-rich green tea extract (EGCG), caffeine, and methyl-xanthine have been reported to promote fatty acid oxidation and inhibit fatty synthesis with anti-obesity effects [15,21]. Overall, scientific evidence confirms the positive effects of ingesting green tea catechins, particularly EGCG [67]. Recently, EGCG-containing green tea extract (EGTE), a new functional food material containing more than 97% of the green tea ingredient EGCG, was developed [68].

Green tea is considered the most representative tea in Korea. Green tea-based foods such as desserts, beverages, and health functional foods have also been widely considered, and events related to green tea have been held continuously in areas where tea is grown. The major strength of this study is the analysis of the risk of abdominal obesity, components of the metabolic syndrome, using data from the KoGES cohort of 10,030 Koreans who routinely consume green tea. With regard to green tea, blood pressure, blood lipids, inflammatory indicators, and obesity have been studied in a variety of ways, but there have been few studies on their association with abdominal obesity. Moreover, this is the first large-scale study to explore the association between green tea consumption and the prevalence of abdominal obesity in the Korean population. Studies on the association between abdominal obesity and several blood indicators support our findings. Recently, as more people have become aware of the efficacy of green tea, several health functional foods using the main ingredients of green tea such as EGCG and EGTE, have been developed. Our findings provide valuable health information that may be of interest to the public.

However, this study has some limitations. Of 3003 women, 1367 were in the abdominal obesity group and 1636 were in the non-abdominal obesity group, creating a similar ratio. However, out of 2875 men, 520 participants were in the abdominal obesity group and 2355 participants were in the non-abdominal obesity group, a difference of four times that may be the cause of disparity in the association between abdominal obesity and green tea consumption between sexes. This is thought to have influenced the lack of statistical power in men. Moreover, a sample from a specific area would not be generalizable in terms of the characteristics of the entire Korean population. Based on the results that differ between men and women, further research on the relationship with green tea intake should be conducted. The effectiveness of green tea depends on the duration and timing of consumption, and the fact that these aspects were not explored in the participants in this study may have affected the results [69]. This study investigated only the consumption of green tea beverages, which limits the present study because the type of green tea leaves and ingredients differ depending on the region, and foods using green tea were not considered [70]. 

## 5. Conclusions

In conclusion, this study found that green tea intake is associated with a reduced risk of abdominal obesity in women. As shown in several studies, the ingredients of green tea are known not only to have anti-obesity effects on blood indicators and lipid profiles, but also to have a beneficial effect on abdominal obesity, a component of metabolic syndrome. Although this study was conducted on middle-aged people, it is expected that adult diseases caused by dietary habits would not be limited to middle-aged people, but would extend many age groups in the future who have an imbalance in nutrient intake and reduced physical activity. Therefore, research that includes various age groups is needed regarding the relationships between green tea, certain nutrients, and physical activities.

## Figures and Tables

**Table 1:** General characteristics of study participants by abdominal obesity in Korean Genome and Epidemiology Study (KoGES).

Variable	Men (n = 2875)			Women (n = 3003)		
	Abdominal Obesity	No Abdominal Obesity	*p*-Value ^1^	Abdominal Obesity	No Abdominal Obesity	*p*-Value ^1^
Participants, n	520	2355		1367	1636	
Age (years)	50.5 ± 8.2	49.9 ± 8.3	0.178	53.5 ± 8.8	48.3 ± 7.7	<0.0001
Area			0.009			<0.0001
Ansung	185 (35.6%)	700 (29.7%)		711 (52.0%)	349 (21.3%)	
Ansan	335 (64.4%)	1655 (70.3%)		656 (48.0%)	1287 (78.7%)	
Education level			0.705			<0.0001
Elementary school or lower	80 (15.4%)	394 (16.7%)		710 (51.9%)	420 (25.7%)	
Middle/High school	320 (61.5%)	1409 (59.8%)		609 (44.6%)	1040 (63.6%)	
College or higher	120 (23.1%)	552 (23.4%)		48 (3.5%)	176 (10.8%)	
Household income (10,000 won/month)			0.297			<0.0001
<100	114 (21.9%)	523 (22.2%)		634 (46.4%)	379 (23.2%)	
100–200	147 (28.3%)	693 (29.4%)		376 (27.5%)	500 (30.6%)	
200–300	105 (20.2%)	523 (22.6%)		203 (14.9%)	393 (24.0%)	
≥300	154 (29.6%)	607 (25.8%)		154 (11.3%)	364 (22.2%)	
Body mass index (kg/m^2^)	27.6 ± 2.0	23.4 ± 2.4	<0.0001	26.5 ± 2.9	22.9 ± 2.3	<0.0001
Alcohol consumption			0.295			<0.0001
None	86 (16.5%)	437 (18.6%)		976 (71.4%)	1107 (67.7%)	
Past	54 (10.4%)	203 (8.6%)		54 (4.0%)	30 (1.8%)	
Current	380 (73.1%)	1715 (72.8%)		337 (24.7%)	499 (30.5%)	
Smoking status			0.122			0.363
None	114 (21.9%)	458 (19.4%)		1297 (94.9%)	1557 (95.2%)	
Past	167 (32.1%)	699 (29.7%)		22 (1.6%)	17 (1.0%)	
Current	239 (46.0%)	1198 (50.9%)		48 (3.5%)	62 (3.8%)	
MET (hours/day) ^2^	23.6 ± 14.8	24.7 ± 15.7	0.017	23.7 ± 14.8	20.1 ± 11.8	<0.0001
Dietary intake						
Total energy intake (kcal/day)	2124.6 ± 726.7	1999.9 ± 620.4	<0.0001	1935.0 ± 730.7	1863.9 ± 636.0	0.005
Protein intake (g/day)	75.2 ± 32.5	69.4 ± 28.1	<0.0001	64.7 ± 29.8	65.0 ± 30.0	0.761
Fat intake (g/day)	39.4 ± 22.7	36.5 ± 21.2	0.008	29.5 ± 19.5	32.0 ± 20.5	0.001
Carbohydrate intake (g/day)	361.8 ± 115.9	343.1 ± 96.7	0.001	348.6 ± 128.7	324.9 ± 103.3	<0.0001
Fiber intake (g/day)	7.3 ± 3.5	6.8 ± 3.1	0.001	7.3 ± 3.8	6.8 ± 3.2	<0.0001
Weekly green tea consumption (cup)	2.7 ± 5.0	2.4 ± 4.2	0.247	1.8 ± 4.3	2.6 ± 4.9	<0.0001
Blood pressure (n = 5872)						
Systolic blood pressure (mmHg)	123.3 ± 15.7	118.0 ± 15.3	<0.0001	120.2 ± 16.6	111.7 ± 16.2	<0.0001
Diastolic blood pressure (mmHg)	84.0 ± 10.1	79.7 ± 10.4	<0.0001	78.9 ± 10.6	73.5 ± 10.2	<0.0001
Biomarkers						
Fasting blood glucose (mg/dL) (n = 5785)	94.2 ± 24.8	89.2 ± 23.4	<0.0001	86.6 ± 23.1	81.7 ± 14.1	<0.0001
Total cholesterol (mg/dL) (n = 5878)	199.5 ± 34.5	191.2 ± 36.1	<0.0001	194.6 ± 37.2	184.9 ± 32.9	<0.0001
HDL cholesterol (mg/dL) ^3^ (n = 5878)	40.1 ± 7.5	44.5 ± 10.0	<0.0001	44.0 ± 9.4	48.4 ± 10.4	<0.0001
Triglyceride (mg/dL) (n = 5878)	215.5 ± 135.3	163.2 ± 107.4	<0.0001	159.6 ± 97.9	122.0 ± 63.4	<0.0001
CRP (mg/dL) ^4^ (n = 5877)	0.28 ± 0.40	0.22 ± 0.41	0.004	0.23 ± 0.38	0.17 ± 0.42	<0.0001
Fasting insulin (uIU/mL) (n = 5786)	8.9 ± 5.5	6.5 ± 3.5	<0.0001	8.7 ± 6.2	7.0 ± 4.3	<0.0001

Data are presented as the mean ± standard deviation (SD) or number (%). ^1^ *p*-values were calculated using the chi-squared test for categorical variables and *t*-test for continuous variables. ^2^ MET, metabolic equivalent of task. ^3^ HDL cholesterol, high-density lipoprotein cholesterol. ^4^ CRP, C-reactive protein.

**Table 2:** General characteristics, biomarkers, and dietary intake of the KoGES Cohort Study participants by weekly green tea consumption (no. of cups).

Variable	Men (n = 2875)					Women (n = 3003)				
	Weekly Green Tea Consumption (No. of Cups)									
	None	<1	1 to <4	≥4	*p*-Value ^1^	None	<1	1 to <4	≥4	*p*-Value ^1^
Participants, n	1099	483	727	566		1137	575	774	517	
Age (years)	52.0 ± 8.9	50.3 ± 8.5	49.1 ± 7.7	47.1 ± 6.5	<0.0001	53.1 ± 8.9	50.7 ± 8.6	48.7 ± 7.9	47.9 ± 7.5	<0.0001
Area					<0.0001					<0.0001
Ansung	439 (39.9%)	185 (38.3%)	178 (24.5%)	83 (14.7%)		557 (49.0%)	238 (41.4%)	168 (21.7%)	97 (18.8%)	
Ansan	660 (60.1%)	298 (61.7%)	549 (75.5%)	483 (85.3%)		580 (51.0%)	337 (58.6%)	606 (78.3%)	420 (81.2%)	
Education level					<0.0001					<0.0001
Elementary school or lower	276 (25.1%)	85 (17.6%)	80 (11.0%)	33 (5.8%)		611 (53.7%)	223 (38.8%)	196 (25.3%)	100 (19.3%)	
Middle/High school	681 (62.0%)	303 (62.7%)	449 (61.8%)	296 (52.3%)		478 (42%)	317 (55.1%)	496 (64.1%)	358 (69.2%)	
College or higher	142 (12.9%)	95 (19.7%)	198 (27.2%)	237 (41.9%)		48 (4.2%)	35 (6.1%)	82 (10.6%)	59 (11.4%)	
Household income (10,000 won/month)					<0.0001					<0.0001
<100	357 (32.5%)	122 (25.3%)	108 (14.9%)	50 (8.8%)		528 (46.4%)	215 (37.4%)	177 (22.9%)	93 (18.0%)	
100–200	336 (30.6%)	166 (34.4%)	219 (30.1%)	119 (21.0%)		311 (27.4%)	185 (32.2%)	224 (28.9%)	156 (30.2%)	
200–300	211 (19.2%)	100 (20.7%)	172 (23.7%)	154 (27.2%)		178 (15.7%)	95 (16.5%)	205 (26.5%)	118 (22.8%)	
≥300	195 (17.7%)	95 (19.7%)	228 (31.4%)	243 (42.9%)		120 (10.6%)	80 (13.9%)	168 (21.7%)	150 (29.0%)	
Body mass index (kg/m^2^)	23.8 ± 3.0	23.9 ± 2.9	24.4 ± 2.7	24.7 ± 2.5	<0.0001	24.5 ± 3.3	24.4 ± 3.1	24.5 ± 3.1	24.6 ± 3.0	0.745
Alcohol consumption					0.204					<0.0001
None	208 (18.9%)	98 (20.3%)	118 (16.2%)	99 (17.5%)		833 (73.3%)	405 (70.4%)	495 (64.0%)	350 (67.7%)	
Past	108 (9.8%)	44 (9.1%)	66 (9.1%)	39 (6.9%)		35 (3.1%)	18 (3.1%)	18 (2.3%)	13 (2.5%)	
Current	783 (71.2%)	341 (70.6%)	543 (74.7%)	428 (75.6%)		269 (23.7%)	152 (26.4%)	261 (33.7%)	154 (29.8%)	
Smoking status					<0.0001					0.382
None	171 (15.6%)	110 (22.8%)	178 (24.5%)	113 (20.0%)		1075 (94.5%)	545 (94.8%)	747 (96.5%)	487 (94.2%)	
Past	283 (25.8%)	147 (30.4%)	243 (33.4%)	193 (34.1%)		17 (1.5%)	7 (1.2%)	9 (1.2%)	6 (1.2%)	
Current	645 (58.7%)	226 (46.8%)	306 (42.1%)	260 (45.9%)		45 (4.0%)	23 (4.0%)	18 (2.3%)	24 (4.6%)	
MET (hours/day) ^2^	24.7 ± 15.7	23.6 ± 14.8	22.4 ± 13.3	20.3 ± 11.9	<0.0001	24.0 ± 15.0	21.4 ± 13.8	20.4 ± 11.8	19.0 ± 10.5	<0.0001
Dietary intake										
Total energy intake (kcal/day)	1972.8 ± 677.9	1912.5 ± 541.4	2039.4 ± 560.5	2191.1 ± 714.0	<0.0001	1825.6 ± 659.5	1850.6 ± 726.4	1969.0 ± 645.0	1993.5 ± 710.2	<0.0001
Protein intake (g/day)	66.6 ± 30.9	63.9 ± 24.5	71.7 ± 23.2	81.7 ± 32.1	<0.0001	59.7 ± 28.8	62.4 ± 34.5	68.9 ± 25.5	72.9 ± 30.4	<0.0001
Fat intake (g/day)	35.1 ± 23.1	32.9 ± 19.2	37.7 ± 17.6	43.3 ± 23.4	<0.0001	27.5 ± 19.2	28.7 ± 22.2	33.9 ± 16.9	36.0 ± 21.9	<0.0001
Carbohydrate intake (g/day)	341.9 ± 107.4	334.4 ± 84.3	347.8 ± 91.4	363.7 ± 109.1	<0.0001	330.1 ± 113.7	331.2 ± 119.2	343.1 ± 114.9	341.6 ± 119.1	0.048
Fiber intake (g/day)	6.6 ± 3.1	6.4 ± 3.2	6.9 ± 2.9	7.6 ± 3.5	<0.0001	6.7 ± 3.4	6.9 ± 3.6	7.3 ± 3.3	7.6 ± 3.7	<0.0001
Blood pressure (n = 5872)										
Systolic blood pressure (mmHg)	120.4 ± 16.5	119.6 ± 15.7	117.2 ± 14.8	117.9 ± 13.7	<0.0001	118.1 ± 17.6	116.1 ± 16.5	113.5 ± 16.2	112.3 ± 16.1	<0.0001
Diastolic blood pressure (mmHg)	80.5 ± 10.8	80.3 ± 10.5	79.9 ± 10.5	81.0 ± 9.8	0.304	77.2 ± 11.0	76.6 ± 10.6	74.7 ± 10.3	74.3 ± 10.6	<0.0001
Biomarkers										
Fasting blood glucose (mg/dL) (n = 5785)	88.2 ± 21.4	89.0 ± 20.4	90.5 ± 22.7	94.4 ± 31.0	<0.0001	84.3 ± 18.4	83.0 ± 18.6	84.2 ± 17.5	83.7 ± 22.0	0.558
Total cholesterol (mg/dL) (n = 5878)	189.7 ± 36.2	190.7 ± 36.0	194.1 ± 34.9	198.6 ± 36.1	<0.0001	190.3 ± 36.0	185.7 ± 34.2	191.1 ± 36.1	188.5 ± 33.3	0.026
HDL cholesterol (mg/dL) ^3^ (n = 5878)	44.1 ± 10.1	43.6 ± 9.6	43.3 ± 9.4	43.3 ± 9.7	0.232	46.2 ± 10.1	45.4 ± 9.8	47.1 ± 10.4	47.0 ± 10.2	0.008
Triglyceride (mg/dL) (n = 5878)	174.3 ± 115.0	168.8 ± 116.6	173.4 ± 117.2	171.8 ± 109.5	0.843	142.3 ± 88.0	142.8 ± 81.9	136.0 ± 83.6	132.5 ± 71.2	0.067
CRP (mg/dL) ^4^ (n = 5877)	0.24 ± 0.50	0.22 ± 0.33	0.22 ± 0.33	0.25 ± 0.38	0.344	0.20 ± 0.41	0.21 ± 0.46	0.20 ± 0.37	0.18 ± 0.33	0.689
Fasting Insulin (uIU/mL) (n = 5786)	6.8 ± 3.8	7.0 ± 4.3	6.9 ± 4.0	7.2 ± 4.4	0.304	8.0 ± 6.7	7.7 ± 4.2	7.6 ± 4.1	7.6 ± 4.7	0.428

Data are presented as the mean ± standard deviation (SD) or number (%). ^1^ *p*-values were calculated using the chi-squared test for categorical variables and ANOVA for continuous variables. ^2^ MET, metabolic equivalent of task. ^3^ HDL cholesterol, high-density lipoprotein cholesterol. ^4^ CRP, C-reactive protein.

**Table 3:** Odd ratios (OR) and 95% confidence intervals of abdominal obesity by green tea consumption in KoGES Cohort Study.

	Weekly Green Tea Consumption (No. of Cups)				
(n = Cases/Total)	None	<1	1 to <4	≥4	*p* for Trend ^2^
All (n = 1887/5878)	(n = 820/2236)	(n = 354/1058)	(n = 430/1501)	(n = 283/1083)	
Crude OR (95% CI)	1.00	0.73 (0.60–0.90)	0.52 (0.43–0.63)	0.45 (0.36–0.56)	<0.0001
Multivariable OR (95% CI) ^1^	1.00	0.89 (0.67–1.18)	0.74 (0.56–0.98)	0.56 (0.41–0.78)	0.001
Men (n = 520/2875)	(n = 200/1099)	(n = 85/483)	(n = 133/727)	(n = 102/566)	
Crude OR (95% CI)	1.00	0.96 (0.73–1.27)	1.01 (0.79–1.28)	0.99 (0.76–1.29)	0.987
Multivariable OR (95% CI) ^1^	1.00	0.85 (0.56–1.29)	0.92 (0.64–1.31)	0.82 (0.54–1.24)	0.439
Women (n = 1367/3003)	(n = 620/1137)	(n = 269/575)	(n = 297/774)	(n = 181/517)	
Crude OR (95% CI)	1.00	0.92 (0.80–1.05)	0.72 (0.64–0.81)	0.65 (0.56–0.74)	<0.0001
Multivariable OR (95% CI) ^1^	1.00	0.92 (0.75–1.12)	0.79 (0.65–0.96)	0.71 (0.57–0.88)	0.004

^1^ Adjusted for age, sex, area, alcohol consumption, smoking, body mass index, education level, household income, metabolic equivalent of task (MET), energy intake, protein intake, fat intake, carbohydrate intake, and fiber intake. ^2^ *p* for trend was calculated using the median of weekly green tea consumption in each category.

## Data Availability

The data underlying the results of our study are not publicly available because of KoGES data policy. Data are available from the Division of Genetic Epidemiology and Health Index, NIH, Korea Centers for Disease Control and Prevention, for researchers who meet the criteria for access to confidential data.
